# Silver Nanoparticles for Water Pollution Monitoring and Treatments: Ecosafety Challenge and Cellulose-Based Hybrids Solution

**DOI:** 10.3390/polym12081635

**Published:** 2020-07-23

**Authors:** Andrea Fiorati, Arianna Bellingeri, Carlo Punta, Ilaria Corsi, Iole Venditti

**Affiliations:** 1 Department of Chemistry, Materials, and Chemical Engineering “G. Natta” and INSTM Local Unit, Politecnico di Milano, Piazza Leonardo da Vinci 32, I-20133 Milano, Italy; andrea.fiorati@polimi.it (A.F.); carlo.punta@polimi.it (C.P.); 2 Department of Physical, Earth and Environmental Sciences and INSTM Local Unit, University of Siena, 53100 Siena, Italy; arianna.bellingeri@student.unisi.it (A.B.); ilaria.corsi@unisi.it (I.C.); 3 Department of Sciences, Roma Tre University of Rome, via della Vasca Navale 79, 00146 Rome, Italy

**Keywords:** silver nanoparticles, nanocellulose, engineered nanomaterials, water monitoring, water treatment, ecosafety, ecotoxicology, eco-design

## Abstract

Silver nanoparticles (AgNPs) are widely used as engineered nanomaterials (ENMs) in many advanced nanotechnologies, due to their versatile, easy and cheap preparations combined with peculiar chemical-physical properties. Their increased production and integration in environmental applications including water treatment raise concerns for their impact on humans and the environment. An eco-design strategy that makes it possible to combine the best material performances with no risk for the natural ecosystems and living beings has been recently proposed. This review envisages potential hybrid solutions of AgNPs for water pollution monitoring and remediation to satisfy their successful, environmentally safe (ecosafe) application. Being extremely efficient in pollutants sensing and degradation, their ecosafe application can be achieved in combination with polymeric-based materials, especially with cellulose, by following an eco-design approach. In fact, (AgNPs)–cellulose hybrids have the double advantage of being easily produced using recycled material, with low costs and possible reuse, and of being ecosafe, if properly designed. An updated view of the use and prospects of these advanced hybrids AgNP-based materials is provided, which will surely speed their environmental application with consequent significant economic and environmental impact.

## 1. Introduction

Engineered nanomaterials (ENMs) are typically defined as materials smaller than 100 nm in at least one dimension, and they have specific surface functionalizations and size-dependent properties, such as high reactivity and large surface-to-volume ratio, that satisfy their wide range of applications, including in sensing [1,2,3,4,5], optics [6,7,8,9,10], energy [11,12,13,14,15], catalysis [16,17,18,19], biotechnology [20,21,22,23,24] and so on. 

Among others, silver nanoparticles (AgNPs) have aroused great interest due to their low cost, synthetic versatility, and chemical-physical properties. For this reason, they are already widely used and present in various commercial products, often as hybrid compounds, that can be responsive to external stimuli [25,26,27,28,29,30]. In fact, although not fully addressed in terms of environmental impact, AgNPs have great marketing value, and their production is expected to reach approximately 800 t by 2025 [17,31,32]. The use of AgNPs is often linked to their antibacterial properties, but in this review, we do not discuss this aspect, for which we refer to the extensive and comprehensive literature already published [29,33,34]. Rather, more recent contributions have revealed the successful use of AgNPs as plasmonic sensors for water pollutants such as heavy metals and organic compounds, and as suitable photocatalysts for promoting the oxidative degradation of the latter, above all dyes and pesticides, which enlarges the field of environmental applications [35,36,37,38].

This review envisages potential solutions of AgNPs in water pollution monitoring and remediation, with particular emphasis on their environmentally safe application. It starts from an (i) updated overview on the incredible features of AgNPs and their easy, cheap and versatile synthesis, moves to (ii) the ecosafety concept by highlighting the relevance of assessing their environmental impact in terms of toxicity for aquatic species achieved by an eco-design approach, and (iii) provides potential advanced hybrids solutions such as cellulose–AgNP composites, which allow synergistic and eco-friendly actions. This review was conceived with the awareness that only a multidisciplinary and integrated approach among biological, chemical, and engineering visions allows a real deepening of the topic of new hybrid ecosafe solutions for water pollution monitoring and treatment and an enrichment that goes beyond the simple collection and proposal of recent articles.

## 2. AgNPs Preparation and Use for Water Pollution Monitoring and Treatment

### 2.1. AgNP Synthesis and Characterizations

The need to improve the performance of sensors in terms of sensitivity, selectivity, reusability and eco-sustainability has led to strong research and development in the field of ENMs [39,40,41,42]. In fact, thanks to the high surface-to-volume ratio, they allow a better interaction with analytes. Furthermore, the possibility of modulating size, shape and functionalization allows their chemical-physical properties to be controlled, such as optical or assembly properties, in order to adapt them to specific needs [43,44,45,46,47,48]. 

AgNPs have been widely used in sensing applications due to their surface properties, such as local surface plasmon resonance (LSPR). LSPR is known to be due to electrons on the surface of noble metal NPs that interact with electromagnetic radiation and produce strong extinction and dispersion spectra, at about 400 nm in the visible spectrum range. This property is incredibly useful for detection. In fact, when an analyte arrives on the surface of AgNPs, the LSPR band can be modified, and very often a change in color can also occur [35,49,50,51,52,53,54,55]. Moreover, AgNPs offer many advantages over other metal NPs (Au, Cu, Li, and Al NPs) due to the possibility of showing LSPR in the visible (vis) and near-infrared regions (NIR) in the range 300–1200 nm [56,57,58]. Ag also has the highest electrical and thermal conductivity among all metals, making it an ideal component for electrical interconnection. When exposed to air, Ag is not oxidized, but forms a silver sulfide film on its surface, which should be more or less transparent to visible light [59,60]. Ag is relatively cheap among metals that support plasmons, and together with its ease of nanofabrication, this determines its usefulness as a metal for plasmonic applications, especially on a large scale.

The LSPR makes AgNPs very interesting for the design of photocatalytic materials active under sunlight radiation. Indeed, AgNPs are already used as efficient photocatalysts for a variety of reactions, including the mineralization of organic pollutants [38,61]. The great advantage of photocatalysis consists of the direct conversion of light energy into chemical energy, thus reducing energy consumption and environmental pollution, in accordance with general rules of sustainable chemistry and green organic synthesis. For these applications, a key point consists of the selection of the proper support for the immobilization of the active catalyst particles. So far, several solutions have been studied, including polymers, cellulose, carbon materials, mesoporous materials, and so on [62,63].

On the other hand, some issues remain. The main concern is related to colloidal instability [33]. Colloidal stability depends on several experimental factors including the type of capping agent and surrounding environmental conditions, such as pH and ionic strength. Many capping agents (for example, thiols, polymers, and surfactants) have been investigated to improve AgNPs suspension stability, preventing their aggregation through electrostatic repulsion, steric hindrance or both. Often the prevalent capping agent is citrate, but citrate-stabilized AgNPs aggregate quickly in standard biological media, such as natural sea water (pH = 7.0), phosphate-buffered saline (PBS pH = 7.2–7.4), and acetate buffer (pH = 5.6). 

Many methods for synthesizing stable AgNPs by chemical, physical and biological processes, using both bottom-up and top-down approaches, have been investigated [64,65,66,67,68]. The well-assessed bottom up approach is based on a wet reduction of Ag^+^ ions in the presence of capping agents. Generally, hydrophilic AgNPs can be synthesized starting from water solution of AgNO_3_ in presence of ligand molecule, and adding a reducing agent, such as sodium borohydride or formic acid [24,68,69,70,71]. 

**Figure 1:**
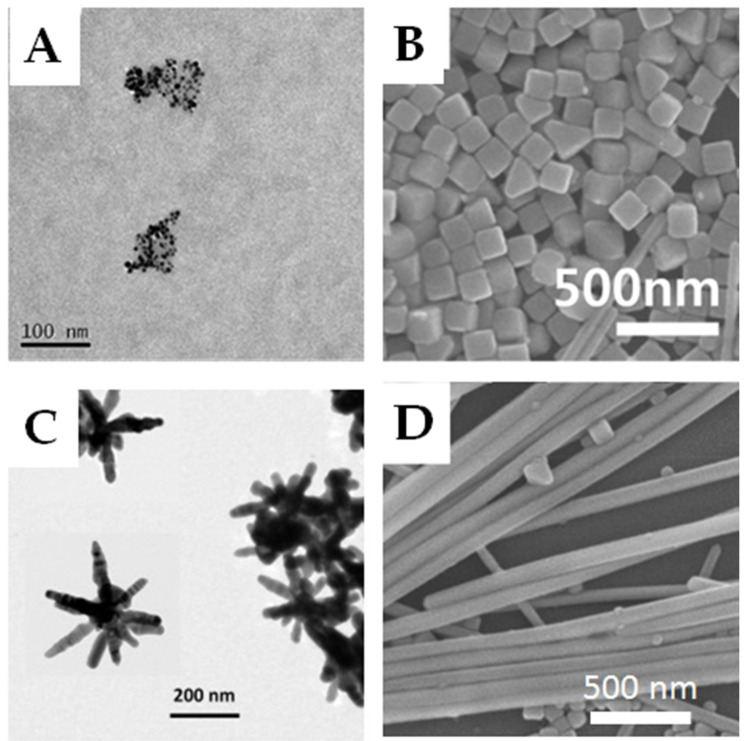
Synthesizing Ag nanostructures: (**A**) spheres, (**B**) cubes, (**C**) stars and (**D**) rods. Reprinted with permission from [68,81,88].

By choosing the appropriate method and experimental parameters for the synthesis it is possible to control shapes and obtain spheres (AgNPhs), cubes (AgNCs), stars (AgNSs) and rods (AgNRs) [72,73,74,75,76,77,78,79,80,81,82,83,84,85,86,87,88,89,90,91,92,93,94,95,96,97,98,99,100]. Some examples with TEM images showing AgNPs with different geometries and shapes are presented in Figure 1 [68,81,88]. 

Among other shapes, the spherical one is the most symmetrical and is the most used because of the easy synthetic control of reproducibility and monodispersity, both being key aspects for ensuring the repeatability of experiments and results. The AgNPhs are synthesized by Turchevich and Shiffrin Brust methods and their modifications, producing the typical LSPR in the range 370–470 nm [26,54,72,73,74,75,76,77]. The well-assessed synthetic studies make it possible to also introduce these silver nanomaterials in nanocomposites, finding application above all in biotechnology and as sensors [55,74,78,79]. 

AgNCs are very promising anisotropic materials. The presence of corners and edges in nanocubes allows an increase in the signal due to the increase in local field. Despite these better properties, the synthesis of AgNCs is still considered difficult, especially as regards the monodispersity quality and reproducibility. In fact, in AgNCs there is material confined by six 100 closed planes, which require precise growth settings for their formation. The experimental parameters that strongly influence these ENMs are the precursor concentrations, the mixing conditions, the temperature and the reaction time. To solve these problems, different methods and techniques have been studied and among them, the microfluidic platforms showed promising perspectives [80,81,82,83,84,85]. 

Star colloidal suspensions of silver (AgNSs) were prepared by chemical reduction of Ag^+^ in two steps and using different reducing and capping agents in each step. The number of star arms varies, with eight generally being the average number. Sometimes these arms can be branched. The AgNSs can have an average diameter from 70 to 700 nm. In general, the tips of the arms display a low sharpness. The extinction spectra the bands are displayed at about 370 nm, and there are extinction background bands at longer wavelengths, where weak maxima are distinguished in the range 650–750. The large extinction background can be attributed to the absorption and scattering emissions produced by the different morphologies of all the existing NPs, integrated by AgNSs bearing different numbers of arms and having different tip sharpness. The mixture of all these factors leads to very different LSPR in the suspension, since a wide range of wavelengths in the visible and near-IR ranges can be covered with various AgNSs shapes and dimensions [86,87,88,89,90,91,92,93].

AgNRs have an anisotropic shape that produces two plasmon bands: the transverse plasmon band due to an electron oscillation along the short axis of the rod, at around 520–550 nm, and the longitudinal plasmon band, in the range 800–1200 nm. Therefore, these ENMs are active in the NIR attracting interest for biomedical application as therapeutic and imaging agents [94,95,96]. Moreover, their aspect ratio (length divided by width) and surface functionalization are easily adjustable by synthetic parameters. There are several AgNR synthesis methods, but the most used are based on seed-based colloidal growth methods, in two steps: in the first step, nucleation is obtained separately by producing seeds which in the second step are added to the growth solution of the nanorods [97,98]. Another widespread technique is based on the oblique angle deposition method (OAD). OAD is a physical vapor deposition method in which the vapor is incident with a large angle (θ > 70°) with respect to the normal surface of substrates, and nanorods or wires are usually formed. By using OAD it is possible to obtain narrower size and more uniform distribution of AgNRs [99,100].

**Figure 2:**
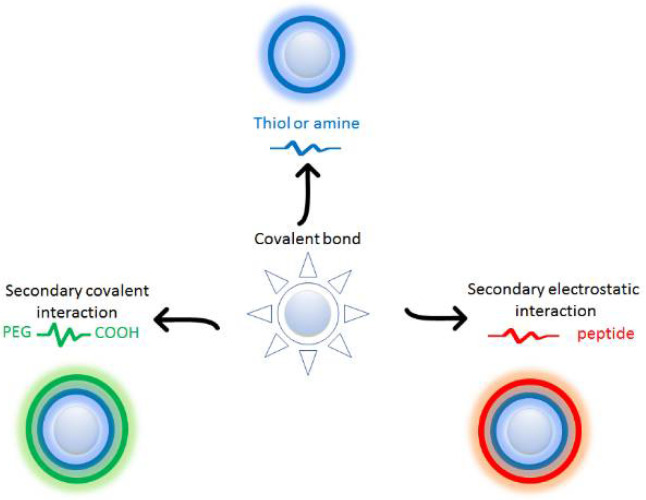
Scheme of main functionalization strategies for AgNPs.

Furthermore, the functionalization and engineering of the Ag surface can be used to guide the aggregation–disaggregation phenomena of ENMs under specific conditions (pH, temperature change or presence of different analytes), leading LSPR band changes. This makes it possible to have a responsive material, capable of interacting with the environment, when properly functionalized. The main strategies for surface functionalization of AgNPs are schematized in Figure 2.

Direct functionalization takes place during synthesis by choosing molecules that stabilize the first clusters of metal particles that are formed by the reduction of Ag(I), generally by sodium borohydride or analogous reducing agents. Usually, these capping molecules exhibit thiol or amine functionalities in which S or N bind covalently to the silver surface. The steric hindrance and any charges present on the cap molecules can induce specific interaction behaviors between particles. After the first functionalization in synthesis, it is possible to resort to a secondary functionalization that engages a second layer of molecules. Electrostatic interactions or covalent bond formation between molecules of the first and second covering layers of AgNPs can be exploited. As for electrostatic interactions, in several cases, the second layer is formed by a peptide, while for the formation of covalent bonds polyethylenglycol (PEG) is often used as ideal coating agent. 

### 2.2. AgNPs for Water Pollution Monitoring and Treatment

The properties and versatility of AgNPs have led them to be widely used in sensor systems. In fact, by changing the type of surface functionalization and by choosing a specific ligand, it is possible to make particles selective to a particular analyte, and by optimizing the degree of surface functionalization, their sensitivity can be improved. The main chemical molecules and capping agents used for direct functionalization of AgNPs during the synthesis of different morphologies are reported in Table 1, together with dimension and applications for water pollution monitoring [26,54,72,73,74,76,77,78,79,80,81,82,83,84,85,86,87,88,101,102,103,104,105,106,107,108]. 

Manivannan et al. [107] developed AgNPhs embedded in an amine functionalized silicate sol–gel matrix, prepared by using different combination of silicate, surfactant and cyclodextrin. The authors observed the blue shift up or quenching of SPR band due to the formation of anisotropic Ag amalgam crystals. The selective sensing of Hg(II) ions by the AgNPh-based sensors in the presence of 500 mM of other environmentally relevant metal ions was verified using spectral and colorimetric methods. Sharma et al. [108] investigated the same behavior for their AgNPhs and developed a simple, label-free, cost-effective, portable, selective, and sensitive colorimetric sensor based on thiol-modified chitosan AgNPhs for the real-time detection of toxic Hg(II) ions in water. In this work, the authors observed an LSPR blue shift in the UV-vis spectra of the solution of Md-Ch-AgNPhs with the addition of the Hg^2+^. This change in the SPR is due to the redox interaction between the Hg(II) ions and AgNPhs, ascribable to the difference in the redox potentials of Hg(II)/Hg couple (0.85 V) and Ag(I)/Ag couple (0.8 V). AgNCs were prepared by Wang et al. using sulfide-mediated polyol method [80]. These AgNCs are active substrates for SERS and allowed the detection of the pesticides paraoxon and thiram. Shkilnyy et al. [106] synthetized AgNRs coated with poly(ethylene glycol) (PEG) covalently attached to their surface. Due to steric repulsion between polymer-modified surfaces, the stability of the nanoparticle suspension was preserved even at high ionic strength (0.1 M NaCl). At the same time, the PEG coating remains sufficiently permeable, allowing surface-enhanced Raman scattering (SERS) from micromolar concentrations of small molecules such as the anticancer drug mitoxantrone (MTX).

Another important feature of AgNPs is their potential as photocatalysts. 

Generally, Ag-based photocatalysts can be classified into two categories: (i) plasmonic photocatalysts based on LSPR effect of AgNPs and (ii) semiconductor photocatalysts based on bandgap excitation of Ag-containing compounds. Upon light irradiation, plasmonic excitation in AgNPs yields hot electrons, while bandgap excitation in Ag-containing semiconductors generates electron hole pairs, which undergo charge separation and transfer, and finally participate in the catalytic reactions. Recent studies have shown that Ag-based visible light responsive photocatalysts could be highly effective for organic pollutant decomposition, bacteria destruction, water reduction and oxidation, and selective organic transformation. In heterogeneous catalysis, free-standing metal NPs usually suffer from low stability due to aggregation. To circumvent this issue, anchoring plasmonic metal photocatalysts on the support materials could enable them to be highly dispersed and easily recycled. 

Some examples of Ag ENMs used for polluted water treatments are reported in Table 2, pointing out AgNP size and supporting the existence of synergic effects in some cases [61,75,109,110,111,112].

A large number of innovative hybrid materials with a synergetic or complementary behavior were obtained using Ag-based photocatalysts. Ullah et al. [109] prepared bismuth vanadate (BiVO4, BV) and Ag/AgO_2_ hybrid nanomaterials with a significant increase (around 28 times) in photoactivity in comparison to pristine BV, as measured by photodegradation of crystal violet and Rhodamine B employing commercial low cost blue LEDs or natural sunlight as photoexcitation sources. The enhanced photoactivity of BV/Ag/AgO_2_ photocatalysts is attributed to the improved adsorption of dyes and extended absorption of visible light by the photocatalysts, and better charge transfer kinetics. Melinte et al. [61] prepared several photocatalysts based on Ag, Au or Au-Ag nanoparticles supported on photocrosslinked organic and these hybrid systems allowed the photocatalytic degradation of 4-nitroaniline. Roy et al. [75] studied the photocatalytic degradation of methylene blue dye in presence of biogenic AgNPs synthesized using yeast (*Saccharomyces cerevisiae*) extract. 

The treatment of water with silver ENMs has also been performed in consideration of their antibacterial action. In fact, AgNPs have wide applications in water disinfection thanks to their well-known antimicrobial activity. Various mechanisms have been proposed in the literature to explain the antimicrobial activity of AgNP which can be traced back to three actions: (1) alteration of membrane properties; (2) damage to DNA/RNA and/or proteins; or (3) release of Ag (I) in the cell cytoplasm. Although in this review the antimicrobial and antibacterial properties of AgNPs are not the focus, how these properties can influence and cause effects on the environment, producing important environmental impacts and causing rebound to their safe application will be discussed in the next paragraph. Today, these are a new ecological challenge. 

## 3. Ecosafety Challenges

### 3.1. Environmental Safety of ENMs

The ecotoxicological implications of ENMs for aquatic and terrestrial organisms have been widely documented and mainly attributed to the peculiar nanoscale properties (i.e., size, shape, surface charges) and their transformation once released in natural ecosystems [113]. 

A large number of bench-scale studies have clearly identified in the nanoscale dimension and surface chemistry the main drivers of cellular uptake by which ENMs can be easily internalized by the cell through different processes (i.e., phagocytosis, endocytosis, direct trans-membrane transport) and exert their toxic action [114,115]. 

A slight change in NP size (agglomeration or aggregation) and/or surface chemistry (i.e., interaction with ionic species and colloidal particles) can significantly affect behavior and exposure dynamics towards living organisms [116,117,118]. Processes such as homoaggregation (i.e., aggregation of ENMs of the same nature) and heteroaggregation (i.e., the aggregation between non-homologous particles), which are dependent on the chemistry of the receiving environmental media (i.e., soil, freshwater and saline) and on the NP functional coatings, may limit their ability to be internalized by the cells as well as their dissolution capabilities [119,120]. On the other hand, processes such as resuspension and disaggregation, luckily occurring in natural water bodies (river, estuaries and oceans) could still make bioavailable a consistent fraction of ENMs to aquatic organisms along the water column or in sediments [121]. Similarly, processes such as oxidation, sulfidation, chlorination and dissolution, more frequent for metal-based ENMs, could significantly affect their environmental behavior and toxicity [29]. 

AgNPs are the most commonly and widely used ENMs in consumer products, mainly due to their biocidal properties which makes them highly efficient anti-microbial agents [1,2,17,122,123,124,125]. A study conducted on two German wastewater treatment plants demonstrated the removal of AgNPs up to 96.4%, with residual concentration ranging from 0.7 to 11.1 ng/L in effluents. However, based on such data, despite the high removal efficiency, the authors estimated a total release in the environment of 33 Kg AgNPs/year for the whole country [126]. For these reasons, AgNPs have stimulated considerable attention in terms of potential environmental risks consequent to their production, usage, disposal and application [127,128,129,130].

### 3.2. AgNP Toxicity to Aquatic Biota

**Figure 3:**
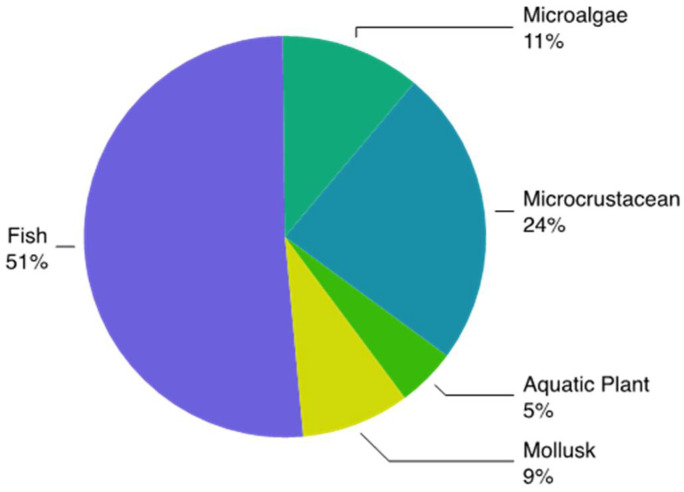
Schematic representation of the distribution of taxa investigated in aquatic ecotoxicity studies with AgNPs up to 2019 (Scopus source). Total number of studies was 282.

The antimicrobial activity of AgNPs is closely linked to the release of Ag^+^ ions [131], which are recognized as one of the most toxic metal ions in the aquatic environment [132]. The use of AgNPs, compared to AgNO_3_, gives a much effective and longer-term antimicrobial activity, due to the continuous and prolonged release of Ag^+^ ions, close to the target organism [133,134]. This feature confers a high potential hazard to the environmental release of AgNPs, since their biocidal properties are not only restricted to microorganisms. In fact, AgNPs are largely documented to be toxic to aquatic biota [30,135] including freshwater [18,39,136] and marine species [34,136,137,138,139,140] (Figure 3). 

As for microorganism, AgNPs’ ecotoxicity to non-target species is mainly imputable to dissolution, even if some studies report that toxicity seems to not be fully explained by dissolved Ag and that a nano-specific toxicity could be involved [128,129]. Additionally, additional effects can be observed as particle size decrease [141,142], even though there is no general consensus on the contribution of each factor [143]. Smaller particles could influence toxicity by providing a larger surface area for the particle’s dissolution [144] or allow cellular internalization [145], resulting in interaction with molecular mechanisms or intracellular dissolution [146].

Numerous studies investigating the toxic effect of AgNPs to non-target organisms are lacking information about particles behavior and dissolution in the media, which is a key element for understanding the mechanism of toxicity and thus their environmental safety. The presence in environmental media of oxidizing species, chlorines or sulfur, could favor the release of Ag^+^ ions and therefore significantly affect toxic action not only towards microbes but in general to all potentially exposed organisms [127]. 

### 3.3. Role of the Surface Coating in AgNPs Ecotoxicity

Among the intrinsic factors influencing the dissolution of the particles, the type of surface coating plays a crucial role, by also influencing particles aggregation state and overall stability in the media [147]. Experimental evidence shows that, in the same conditions, AgNPs with different types of coating result in different toxicities [18,129,145,148]. A safe design strategy was proposed by Pang et al. [130], who demonstrated, both in vitro (Hepa1c1c7) and in vivo (mice), that biodistribution, intracellular localization and toxicity of AgNPs were significantly affected by NP surface coatings and in particular by the presence of positive surface charges (BPEI AgNPs > Citrate AgNPs = PVP AgNPs > PEG AgNPs). 

Some coating agents can prevent the dissolution of the particles, either by closely bind AgNPs surface and preventing Ag^+^ ions to solubilize or by excluding oxidizing agents to come in contact with the particle’s surface, preventing the particle’s oxidation and consequent dissolution [70]. That is the case of molecules with a high reduced sulfur content that bind metal ions with high affinity [149]. The toxicity of AgNPs for both microbes and non-target species has been shown to decrease following the addition of molecules rich in thiol groups in solution, such as natural organic matter or cysteine [18,150,151,152].

Several studies have assessed the toxicity of sulfur functionalized AgNPs, and always reported a reduction in toxicity compared to pristine AgNPs. Levard et al. [153] reported that, as the level of sulfidation of AgNPs increased, the toxicity to four different model organisms (fish embryos, worm and aquatic plant), as well as the dissolution of the particles, decreased. Another study reported that AgNPs functionalized either with cysteine or glutathione showed a lower toxicity to the microcrustacean *Daphnia magna* compared to reported literature data on differently coated AgNPs [154]. 

### 3.4. Ecosafe by Design Approach

**Figure 4:**
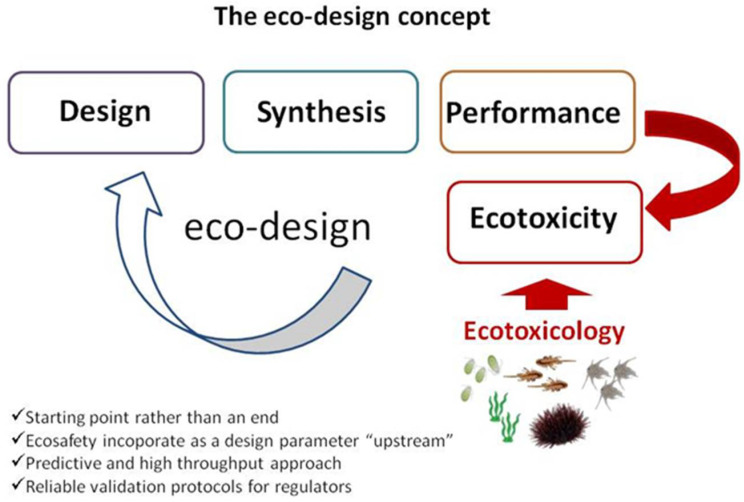
The eco-design concept based on ecotoxicological risk assessment as a tool for eco-design achievement [158].

Ecosafety has begun to be included in a risk assessment framework to support policy-makers, legislators and stakeholders with the aim of limiting any risk associated with their use and application [29,155]. To achieve environmental safety, the new strategy widely adopted in nanomedicine to design safer materials has been recognized to be successful in order to disregard those undesirable properties which can be hazardous for humans and the environment [32]. A similar concept was recently adopted in the design of ENMs for environmental application as for instance in pollution remediation (nanoremediation), in which environmental safety and efficacy towards removal of pollutants from an environmental setting will be incorporated in the material design process (eco-design) [28,32,156,157]. Removal efficacy and ecotoxicity are tested in parallel in order to obtain best adsorption performances with no risk for the exposed organisms (Figure 4). 

By reducing nanomaterial uncertainties and environmental and human risks, an eco-design strategy could support regulatory requirements, boost circular economy and act as a further driver for market development of the nanoremediation sector.

As an example, in line with this strategy, a recent contribution in the field was made by selecting ecosafe batches of cellulose-based nanosponges (CNS) for heavy metal removal from seawater obtained by an eco-design strategy [147]. A trial-and-error-like process in which direct effects on aquatic organisms of CNS, evaluated by an ecotoxicological approach, aids the formulation of CNS in a stepwise fashion (i.e., by testing single components and synthesized materials at the final stage) along with the synthetic procedure and it has been proven to be successful in selecting the most environmental safe CNS formulation [156,158]. 

By testing two aquatic trophic levels, planktonic and filter-feeder benthic species, as potential biological targets of CNS, the eco-design strategy allows to select those ecologically safer material properties to avoid any potential ecological risks consequent to their environmental application. A more recent contribution clearly demonstrated that effect-based tools as biological responses in exposed organisms can be used to validate adsorption efficacy of CNS towards Zn removal from seawater and prevent potential ecological risks due to their application [159,160].

**Figure 5:**
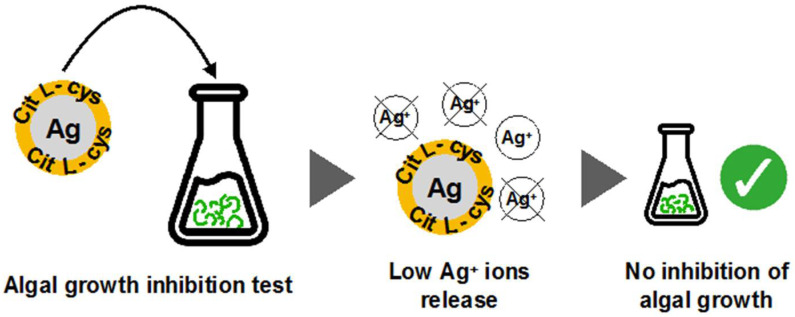
Testing of AgNPcitLcys for algal toxicity resulted in low release of silver ions and no inhibition of algal growth, from both fresh- and marine waters [54].

Furthermore, the same eco-design strategy was recently applied with a newly synthesized batch of AgNPs functionalized with citrate and L-cysteine (AgNPs@Cit/L-cys), and thus was able to selectively adsorb mercury (Hg^2+^) from water. This peculiar functionalization apparently prevents the release of Ag^+^ confirmed by the absence of toxicity towards freshwater and marine microalgae (*Raphidocelis subcapitata* and *Phaeodactylum tricornutum*) (Figure 5). 

The high adsorption capability towards Hg^2+^coupled with no risks for aquatic species clearly support their application for in situ remediation. For more details refer to Prosposito et al. [54]

Therefore, a more comprehensive understanding of the role of AgNPs surface coatings, not only in driving their behavior in environmental settings but also their ability to be taken up by aquatic organisms, is becoming mandatory for supporting ecosafety of ENM for environmental application. In this regard, the need to predict realistic exposure scenarios as for instance the in situ and ex situ applications stimulates the adoption of an effect-based approach which will mimic environmental exposure conditions [54,159].

## 4. Cellulose Doped with AgNPs: A Synergic Solution 

### 4.1. A Sustainable Solution for AgNP Immobilization

A valuable route to overcoming the concerns related to the direct use of AgNPs, due to the potential toxicity of these systems, consists of their immobilization on proper supports. This approach, which would allow the limiting of NP migration and even the improvement of remediation action by preventing their agglomeration due to surface energy, takes inspiration from technologies developed to transfer to textiles the most investigated property of AgNPs, namely the antimicrobial activity. For this reason, biopolymers have been widely considered as ideal substrates for AgNP binding [158,161].

While the first applications of this approach were in the field of textile materials for medical, health care, and hygienic uses, researchers envisioned the possibility of also applying AgNPs/biopolymer composites for the safe sanitization of drinking water [162,163]. 

In consideration of the previously discussed adsorption/degradation action of silver nanoparticles, this technology has more recently been extended to the design of devices, filters, and membranes for wastewater decontamination. The knowledge achieved in the field of textile functionalization fits well with this specific target in terms of materials of choice for supporting the nanoparticles. In fact, biopolymers such as cellulose, starch-derived dextrin, and chitosan are becoming more and more attractive as ideal building blocks for the production of smart materials for water treatment [164]. Polysaccharides allow the combination of their renewable and biodegradable nature with a negligible toxicity, meeting the ecosafety premise necessary to follow the eco-design of sustainable solutions for the environmental monitoring and remediation, as discussed in Section 3 [32].

**Figure 6:**
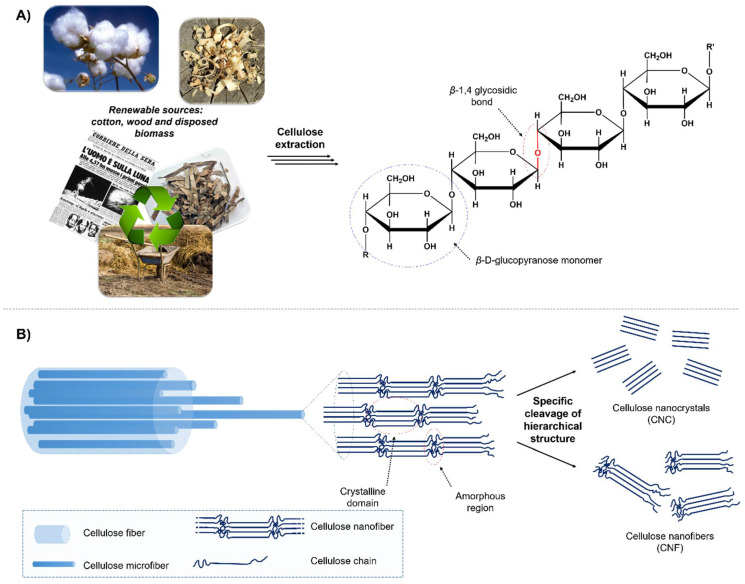
(**A**) Cellulose sources and polymer structure; (**B**) hierarchical structure of cellulose nanofibers.

Cellulose is the most abundant biopolymer on Earth, as it can be extracted from a wide range of renewable sources, such as cotton, wood, and disposed biomass, including agricultural waste and recycled paper (Figure 6a) [156]. Cellulose fibers present a high-mass molecular structure, formed by the repetition of a huge number of β-d-glucopyranose monomers linked together by β-1,4 glycosidic bonds [165]. This structure favors the synthesis and binding of nanoparticles, acting as stabilizing and capping agent.

Moreover, it is possible to cleave the hierarchical structure of cellulose fibers by following different mechanical or chemical treatments, producing nanocellulose (NC) in the form of nanocrystals (CNC) or nanofibers (CNF), the latter presenting crystalline domains alternating with amorphous regions (Figure 6b) [166,167,168]. Moving to a nanoscale dimension it is possible to increase significantly the surface area and the biopolymer reactivity, opening the way to a selective functionalization of NC, in order to introduce additional moieties for enforcing nanoparticle immobilization. The introduction of new much more environmentally and economically sustainable protocols, such as the 2,2,6,6-tetramethylpiperidine 1-oxyl (TEMPO)-mediated oxidation [169,170] and the enzymatic (endoglucanase) pretreatment [170,171,172], favored the scaling up of NC production up to the order of hundreds of kilograms per day (http://www.tappinano.org, 2019).

Finally, cellulose- and even more nanocellulose-based materials have been reported to be able to efficiently adsorb a wide range of transition metal ions and organic dyes and pollutants [158,173,174,175]. As a consequence, it is possible to combine, in the same composite, the high adsorption efficiency of NC with the catalytic or photocatalytic action of AgNPs for promoting the degradation of contaminants in water.

### 4.2. Cellulose Doping with Pre-Formed AgNPs 

The simplest approach for the synthesis of AgNPs/cellulose composites consists of the use of commercially available NPs or their pre-formation in a separate batch. In this case, cellulose doping occurs by contacting the NP aqueous dispersion with the polymeric support. Sehaqui and coworkers reported the synthesis of CNF-based filters and the capture of a wide range of nanoparticles, including silver ones, by simple filtration [176]. CNF were first functionalized in part with quaternary ammonium moieties using 2,3-epoxypropyl trimethyl ammonium chloride, and in part with carboxylic groups by means of succinic anhydride. The filter was prepared by freeze-drying a homogeneous dispersion of the two typologies of CNF, providing a 2D sheet-like microporous structure, in line to what reported for similar systems [176,177,178]. 

Gold and silver nanoparticles were fixed thanks to the electrostatic interaction between the positive surface of the filter and the negative zeta potential of the NPs. Such doped filters were used for the adsorption and reduction of 4-nitrophenol and methylene blue (MB), chosen as model water contaminants.

With a similar approach, Ismail et al. designed a naked eye sensor for Hg^II^, Cr^VI^, and ammonia in aqueous solution. They proceeded by dropping on a cellulose filter paper a colloidal solution of AgNPs, freshly prepared by using an aqueous leaf extract of *Convolvolus cneourm* to reduce Ag^+^ ions and stabilize the resulting particles [179].

### 4.3. Cellulose Doping with AgNPs Generated in Situ

NPs can be also synthesized in situ and directly fixed on cellulose, which represents the preferred approach in many studies. As an example, a recent paper describes the synthesis of Ag_3_PO_4_ particles from AgNO_3_ in the presence of NC sheets, following an ion exchange method [180]. The Ag_3_PO_4_/NC composite results in being particularly effective at promoting the sunlight-induced photodegradation of MB and methyl orange (MO), both in deionized water and in a more complex wastewater matrix.

**Figure 7:**
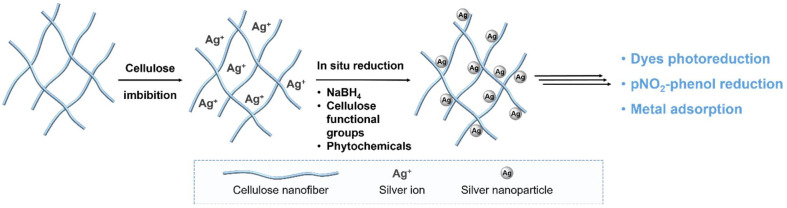
Process scheme for the in situ production of AgNPs/cellulose composites for water treatment.

More commonly, the immobilization of AgNPs on the cellulose support occurs by following the scheme depicted in Figure 7. 

The combination of different and complementary analytical techniques is necessary to confirm nanoparticle formation (XRD), bonding on the cellulose support by involving hydroxyl groups (FTIR), and homogeneous dispersion of nanoparticles on the polymeric matrix (SEM, TEM).

The standard procedure implies the impregnation of the polysaccharide substrate with an aqueous solution containing Ag^+^ ions, usually provided in the form of AgNO_3_ salt, and the consequent reduction of the metal ion with a proper reductant agent, like NaBH_4_. Following this approach, Zia’s group recently described an efficient adsorption process for the removal of metal ions from aqueous solutions, including wastewater, using AgNPs coated cotton cellulose as sorbent unit [181,182].

As the use of chemical reducing agents generates concerns, due to waste management issues, which in turn have a negative impact on the eco-design of final composites, in recent years the bioreduction solution has attracted more and more attention, being considered the safest and most convenient green option for AgNPs formation in situ. This consists of the use of phytochemicals like polyphenols, flavonoids, and alkaloids, extracted from different natural sources, as Ag^+^ reductants. Albukari and coworkers have proposed the use of *Duranta erecta* leaves’ extract for synthesizing AgNPs directly on cellulose polymer paper [20]. Das’ group considered the *Hibiscus sabdariffa* shrub as a source of both nanocellulose from stem and reducing agents from flowers and leaves [183]. In both examples, the final composites were applied as effective photocatalysts under visible light radiation for the reduction of nitrophenols and a wide range of organic dyes in wastewater treatments. In a recent paper, Zhang and co-workers also proposed ascorbic acid as nontoxic reducing agent [184]. In this case, nanocomposites were synthesized through a double vegetable oil-based microemulsion method, using an ionic liquid (1-ethyl-3-methylimidazolium acetate) as a polar phase. Following this route, it was possible to simply control nanoparticle sizes and their homogeneous dispersion. The resulting material was successfully tested as catalyst for the reduction of nitrophenols in the presence of NaBH_4_.

AgNP formation in situ could also be achieved without requiring the addition of external reductant agents. In 2013, Dong and coworkers reported the binding of Ag^+^ ions onto TEMPO-oxidized CNF, followed by a slowly reduction of the cations by means of the hydroxyl groups of the glucopyranose units [185]. More recently, Pawcenis et al. deeply investigated this reduction mechanism [186]. Since the presence of aldehyde groups deriving by a partial oxidation has been excluded after both solution-state ^1^H-NMR and solid-state ^13^C-NMR, and FTIR analysis, they hypothesized that carbohydrates could play a similar role to diols, due to the high presence of vicinal hydroxy groups. Polyols can dehydrate to the corresponding unstable aldehydes, whose oxidation to carboxylic acids provides the electrons for the cation reduction.

Chook’s group adopted this approach for the design of a porous AgNPs/CNF aerogel nanocomposite, which revealed superb performances in detecting and catalyzing the degradation of rhodamine B from water solutions, in the presence of NaBH_4_ [112,187]. Similarly, Han et al. proposed the reductant-free synthesis of AgNPs-doped cellulose microgels for mediating the reduction of nitrophenols, always by means of NaBH_4_ [188].

As stated before, one of the advantages in using NC supports consist into the significant increase of the reactive surface area, which also implies the possibility to functionalize further the cellulosic template in order to guarantee a stronger and more homogeneous binding of silver nanoparticles. A first approach could consist into simply adsorb functional moieties onto cellulose backbone. For example, An et al. showed how the adsorption of a surfactant (hexadecyl-tetramethylammonium bromide) onto CNC favors the binding and dispersion of AgNPs on the support, providing a device with a higher catalytic activity towards the reduction of 4-nitrophenol to 4-aminophenol, compared to AgNPs/CNC standard systems [189]. 

More commonly, functionalization occurs by covalently fixing new binding moieties. To design new membrane supports for water treatment, Nicolaus’ group prepared amino-modified nanocellulose, using 3-aminopropyltriethoxysilane as a grafting agent, on which silver nanoparticles were fixed by in situ reduction with NaBH_4_. The membrane performance was tested by measuring the total organic carbon (TOC) abatement from wastewater [190]. A greener approach to fix AgNPs on CNC was described by Tang and co-workers [191]. This consisted of the cellulose coating with mussel-inspired polydopamine (PDA), which also played the role of reducing agent for Ag^+^ ions. The resulting AgNPs/PDA/CNC composite showed a core–shell structure decorated with nanoparticles. This system resulted in be six times more efficient in catalyzing the reduction of 4-nitrophenol, compared with the AgNPs/CNC model. The catalytic efficiency was even more accelerated by combing this CNC/PDA hybrid with *β*-cyclodextrin [191].

## 5. Conclusions and Perspectives

AgNPs’ increasing integration into environmental applications, including water pollution monitoring and treatment, is raising concerns about their environmental impact, so much so that an eco-design strategy is proposed that combines better performance with the absence of risks for ecosystems. The grafting of AgNPs onto purposely selected polymers represents a potential solution for overcoming both ecosafety concerns and nanoparticle coalescence. In this context, with this contribution, starting from an overview of the recent preparations of AgNPs and their uses, we indicate in the (AgNPs)–cellulose hybrid materials potential solutions leading to efficient and eco-friendly materials for the monitoring and treatments of water pollution. (AgNPs)–cellulose hybrids have the double advantage of being easily produced using recycled material, with low cost and possible reuse, and of being eco-safe when properly designed. We envision that the development of these hybrid materials by following an eco-design approach would represent a winning strategy for valorizing AgNP-based nanotechnologies for water pollution monitoring and treatment.

## Figures and Tables

**Table 1:** Main morphologies reported with dimension, surface functionalities and application for water pollution monitoring and treatments.

Shape	Dimension (nm)	Surface Functionalization	Detection and Monitoring of Pollutant	Ref.
**Spheres**				
	10–15	chalcone carboxylic acid	Cd(II)	[72]
	5–8	sodium 3-mercapto- 1-propanesulfonate	Co (II); Ni (II)	[26]
	-	methyl cellulose	Cu (II)	[74]
	10–20	Riboflavin	Hg (II)	[79]
	5–8	Citrate/L-cysteine	Hg (II)	[54]
	10–15	ciclodextrin	Hg (II)	[107]
	6	Thiol terminated chitosan	Hg (II)	[108]
	20	polyvinyl alcohol	Pb(II)	[73]
	9–10	gluconate	Pb(II)	[76]
	2	polyethyleneimine (PEI)	p-nitrophenol	[101]
	5–10	thioglycolic acid	6-benzylaminopurine	[77]
	-	citrate+hexapeptide	Malathion ^1^	[78]
	8–10	cyclen dithiocarbamate-	Thiram; paraquat ^1^	[102]
**Cubes**				
	100	Poly(vinylpyrrolidone)	Paraoxon; thiram ^1^	[80]
	200	glycolaldeyde	-	[81]
	90–100	cetyltrimethylammoniumcloride	-	[82]
	40–80	Poly(vinylpyrrolidone)	-	[83]
	200–300	polyaniline	hemoglobin	[84]
	60–100	hexamine	Bis phenol	[85]
**Stars**				
			glucose	[87]
	50–150	Lauryl sulfobetaine	Melamine	[86]
	180–250	Citrate/hydroxylamine	Congo Red	[88]
**Rods**				
	150–250	--	antibiotic	[103]
	--	Cy5-ssDNA	Hg(II)	[104]
	30–200	--	Polyclorinated biphenyls	[105]
	10–20	poly(ethylene glycol)	mitoxantrone	[106]

^1^ Pesticides.

**Table 2:** AgNP-based hybrid systems used for water pollution treatments.

AgNPs Size (nm)	Support	Treatment	Ref.
5–10	photocrosslinked matrix	nitroderivates	[61]
10–20	BiVO_4_	crystal violet; Rhodamine B	[109]
10–30	sulfonated graphene/TiO_2_	Rhodamine B; Methyl Orange; 4-nitrophenol	[110]
10		Methylene blue	[75]
5–10	TiO_2_	Methyl Orange	[111]
100	cellulose nanofibrils	Rhodamine B;	[112]
